# Transcranial microtesla magnetic fields suppress neuroinflammation and neuronal oxidative stress burden

**DOI:** 10.1016/j.isci.2025.114425

**Published:** 2025-12-15

**Authors:** Nhu Nguyen, Nathan R. Brady, Greg A. Timblin, Kevin M. Tharp, Blake T. Gurfein

**Affiliations:** 1 Fareon, Inc, 135 Mississippi St, San Francisco, CA 94107, USA; 2 Inapill, Bakar Labs, 2630 Bancroft Way, Berkeley, CA 94704, USA; 3 Sanford Burnham Prebys Medical Discovery Institute, 10901 N Torrey Pines Rd, La Jolla, CA 92037, USA

**Keywords:** health sciences, therapeutics, biological sciences, molecular biology

## Abstract

Neuroinflammation is a major driver of neurodegenerative and psychiatric disease, yet current therapies have limited brain penetration and efficacy. We investigated microtesla magnetic therapy (MMT), a brief transcranial exposure to time-varied electromagnetic fields (TV-EMFs), as a noninvasive approach to modulate neuroimmune inflammation. In human peripheral blood mononuclear cells, MMT suppressed lipopolysaccharide (LPS)-induced TNFα and IL-1β release and reduced NF-κB activation in monocyte and macrophage lines. In rats with intracerebral LPS injection, a model of progressive neuroinflammation, repeated head-localized MMT markedly decreased microgliosis, astrogliosis, and lesion size. In a neuron-immune cell model, MMT reduced cytokine-driven and paraquat-induced oxidative stress, producing both indirect and direct neuroprotection lasting up to 48 h. Collectively, these findings validate transcranial MMT as a promising, noninvasive biophysical therapy for neuroinflammatory conditions. Both acute and repetitive TV-EMF protocols delivered robust anti-inflammatory, antioxidant, and neuroprotective effects, demonstrating the therapeutic potential of precisely modulated EMFs to safely manage neuroinflammation.

## Highlights

• MMT suppresses NF-κB signaling and reduces TNFα and IL-1β in human immune cells
• Transcranial MMT reduces microgliosis, astrogliosis, and tissue degeneration in rats
• MMT lowers cytokine- and toxin-driven oxidative stress, providing neuroprotection
• Noninvasive MMT produces sustained suppression of brain neuroinflammation *in vivo*

## Graphical abstract

**Figure:**
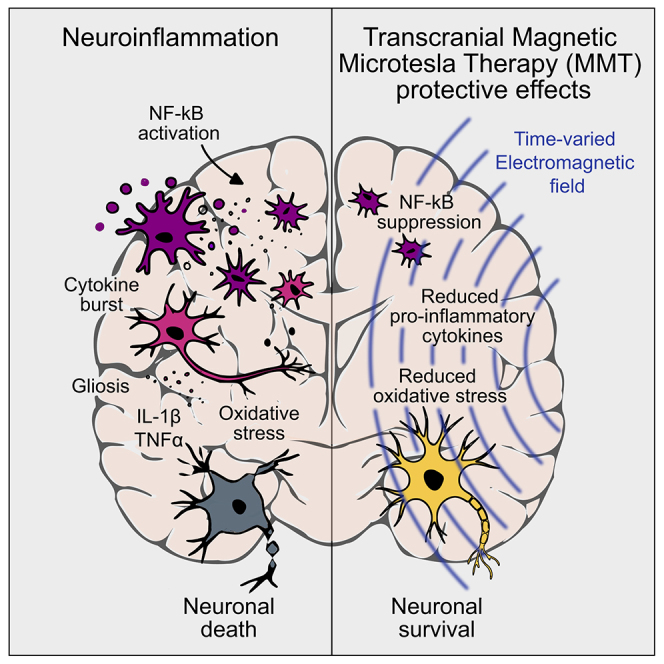

## Introduction

While transient neuroinflammation is crucial for central nervous system (CNS) defense and repair mechanisms,^1^ excessive or sustained activation contributes to a wide range of neurological diseases and psychiatric disorders,^2^^,^^3^ including Alzheimer’s and Parkinson’s disease,^4^ traumatic brain injury (TBI),^5^ bipolar disorder,^6^ and autism.^7^ Detrimental neuroinflammation can be initiated by glial inflammatory receptors which detect danger signals, triggering intracellular cascades that produce pro-inflammatory cytokines and reactive oxygen species (ROS).^8^ These inflammatory pathways can establish amplifying feedback loops which lead to reduced cerebral blood flow, blood brain barrier (BBB) permeability,^9^ mitochondrial damage, chronic neuronal oxidative stress and dysfunction, and consequent neurodegeneration.^10^ Central to neuroinflammation is nuclear factor-kappa B (NF-κB)-mediated glial production and release of key inflammatory cytokines.^11^ Following stimuli such as lipopolysaccharide (LPS), NF-κB induces transcription of inflammatory cytokines, including TNFα and inactive pro-IL-1β.^12^ Subsequent activation signals, such as ATP or mitochondrial ROS,^13^ activate inflammasomes which process and release active IL-1β and IL-18 and initiate pyroptosis, an inflammatory mode of programmed cell death,^12^^,^^13^^,^^14^ while simultaneously promoting glial activation and gliosis.^15^^,^^16^

Despite increasing mechanistic understanding of neuroinflammation processes, effective therapeutic strategies to modify these processes remain limited due to the complexity of brain immunity and challenges in drug delivery across the BBB. Biophysical interventions using electromagnetic fields (EMFs) can be delivered transcranially to both superficial and deep brain structures,^17^ offering a compelling noninvasive strategy to locally modulate neuroimmune pathways, independent of the BBB. If EMF regimes could be applied to impact neuroimmune function, this type of localized therapy would have the potential to provide focused control over neuroinflammatory responses in brain regions without systemic drug exposure, thereby overcoming the existing therapeutic barriers and limiting off-target biological responses.

At the molecular level, TV-EMFs, also known as pulsed EMFs (pEMFs), can be utilized to reduce levels of pro-inflammatory cytokines, including TNFα and IL-1β,^18^ modulate ROS-associated signaling,^19^ and activate the NRF2 antioxidant pathway.^20^^,^^21^^,^^22^ Clinically, EMF-based interventions reduce neuronal inflammation and improve recovery following CNS injury.^23^^,^^24^^,^^25^^,^^26^ However, while apparently effective in certain clinical settings, the cellular and molecular signaling mechanisms underlying beneficial EMF effects, particularly in immune cells and the CNS, remain incompletely understood, which limits optimization of therapeutic protocols for physiological benefit.

In this study we investigated the potential of a TV-EMF exposure protocol, termed microtesla magnetic therapy (MMT), which uses a pulsed high-frequency carrier wave as a noninvasive intervention for neuroinflammation. We focused on both *in vitro* and *in vivo* MMT effects on immune cell and neuronal responses under inflammatory conditions. We report that MMT significantly suppressed LPS-induced pro-inflammatory cytokine production in primary human peripheral blood mononuclear cells (hPBMCs), suppressed LPS-activated NF-κB signaling in monocyte and macrophage cell lines, and reduced LPS-sensitization of the inflammasome-driven mode of programmed cell death, pyroptosis.^27^

We then tested whether these anti-inflammatory effects translated to neuroprotection *in vivo*. Using localized, transcranial MMT administration in an intracranial LPS-induced rat model of progressive neuroinflammation, we observed that MMT markedly reduced microgliosis, astrogliosis, and brain lesion size. Furthermore, *in vitro* analysis of neuroimmune interactions revealed that MMT resulted in indirect neuroprotective effects via reduced immune cell inflammatory cytokine production as well as direct neuroprotective effects through increased neuronal oxidative stress handling, with effects persisting up to 48 h post MMT. Collectively, these findings establish MMT as a biophysics-based therapeutic modality with broad efficacy in restraining neuroinflammatory progression when administered either prophylactically or therapeutically. The dual-compartment anti-inflammatory and neuroprotective effects, coupled with sustained post-treatment benefits, position transcranial EMF as a promising platform technology for both acute and chronic neuroinflammatory conditions.

## Results

### MMT suppressed LPS-induced cytokine secretion in hPBMCs

**Figure 1:**
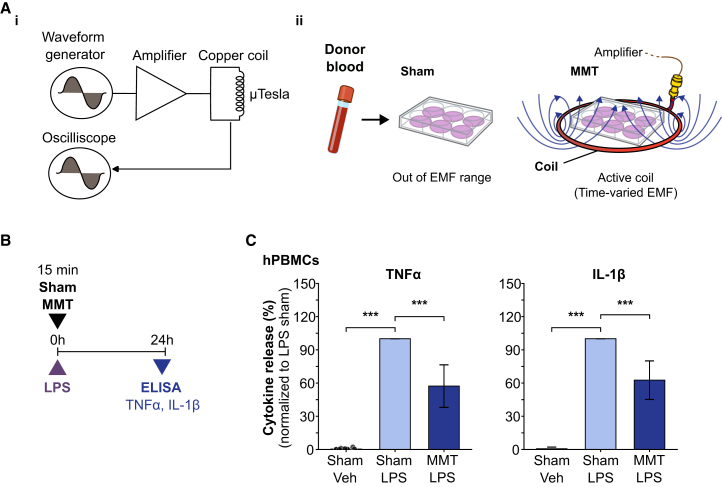
MMT inhibited LPS-induced cytokine secretion in hPBMCs (A) Schematics of (i) the MMT device, comprising a waveform generator, amplifier and coil used to deliver a time-varied EMF exposure and (ii) experimental setup showing MMT treatment (15 min) of a hPBMCs in a 6-well plate positioned within a copper coil. The sham 6-well plate was positioned adjacent to the coil, out of EMF range. (B) Schematic of experimental protocol. hPBMCs were treated with control (DMSO) or LPS (100 ng/mL), with or without MMT, supernatants were collected at 24 h for ELISA analysis. (C) TNFα and IL-1β cytokine levels in culture supernatants measured by ELISA and normalized to the LPS+Sham control (100%). Data from 11 independent donors. Data are presented as mean ± SD. Statistical significance was determined by unpaired *t* test. ∗*p* < 0.05, ∗∗*p* < 0.01, ∗∗∗*p* < 0.001.

For this study we developed an MMT device composed of a signal generator, amplifier, and a tuned coil to noninvasively deliver 15 min exposures of a high-frequency carrier wave to hPBMCs from multiple donors (Figure 1A). To investigate the impact of MMT on inflammatory signaling, hPBMCs were treated with 100 ng/mL LPS to induce cytokine production and release and immediately submitted to either MMT or a sham treatment. Cytokine levels were determined by ELISA of supernatants collected from LPS-stimulated hPBMCs after 24 h. LPS treatment significantly increased pro-inflammatory cytokines, with TNFα increasing by 210-fold and IL-1β increasing by 118-fold (Figure 1C). Notably, MMT significantly suppressed the LPS-induced pro-inflammatory response, reducing the production of TNFα by 43% and IL-1β by 37%.

### MMT suppressed LPS-induced inflammatory signaling

**Figure 2:**
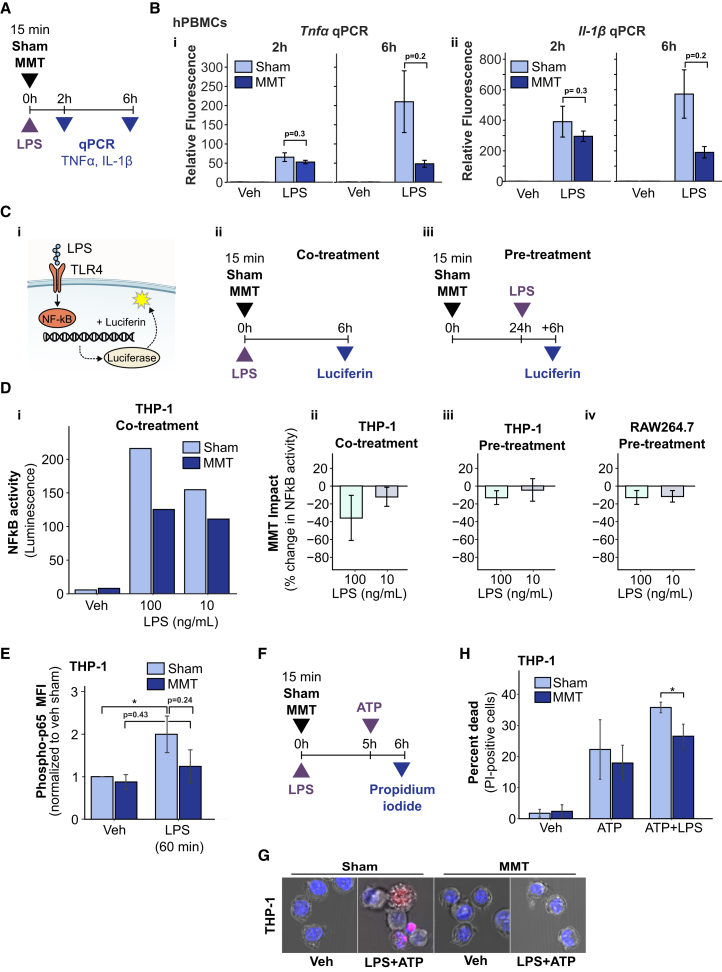
MMT suppressed LPS-activated inflammatory signaling in mononuclear immune cells (A) Schematic of experimental protocol. hPBMCs were treated with vehicle control (DMSO) or LPS (100 ng/mL), with or without MMT, and cells were collected at 2 and 6 h for qPCR analysis. (B) mRNA levels for (i) *Tnfα* and (ii) *Il-1β* measured by qPCR for hPBMCs treated as in (A). Data from 2 independent donors. (C) (i) Schematic of LPS-induced NF-κB luciferase reporter, stably expressed in THP-1 and RAW264.7 cell lines, and experimental protocols for (ii) MMT co-treatment of THP-1 cells and (iii) MMT pre-treatment of THP-1 and Raw264.7 cells. (D) NF-κB luciferase activity in MMT-treated cells. MMT co- and pre-treated THP-1 cells and pre-treated RAW264.7 cells were treated for 6 h with vehicle (DMSO) or LPS (100 or 10 ng/mL), and luciferase activity was measured. (i) Representative data showing MMT suppression in co-treated THP-1 cells. Quantification of NF-κB suppression by MMT in (ii) co-treated and (iii) pre-treated THP-1 and RAW264.7 cells. (E) MMT reduced LPS-induced NF-κB p65 phosphorylation. THP-1 cells were treated with MMT or sham and stimulated with vehicle (DMSO) or LPS (100 ng/mL). Quantification of phosphorylation of p65 at Ser536 by immunofluorescence at 60 min post-LPS stimulation. (F) Schematic of LPS-sensitized pyroptosis in the THP-1 cell line. (G) Representative images of THP-1 cells under pyroptotic conditions. Maximum-intensity projections of z-stacks of sham and MMT-treated THP-1 cells primed with LPS (100 ng/mL) for 5 h, followed by 1 h treatment with ATP (5 mM). Hoechst (blue) labels nuclei, and propidium iodide (PI, red) labels cells with loss of membrane integrity. 63× objective; scale bars, 5 μm. (H) MMT reduced inflammatory cell death in THP-1 cells. Sham and MMT THP-1 cells were primed with LPS (100 ng/mL) for 5 h, treated with ATP (5 mM) for 1 h, and cell death was measured by counting PI-positive cells. Data are presented as mean ± SD. Statistical significance was determined by unpaired *t* test. ∗*p* < 0.05, ∗∗*p* < 0.01, ∗∗∗*p* < 0.001.

To identify the mechanisms underlying suppression of pro-inflammatory cytokine release, we examined the effect of MMT on LPS-induced priming and activation events that drive cytokine production and release.^12^ Transcriptional responses were measured by qPCR, sampling at 2- and 6-h timepoints following simultaneous exposure to the co-treatment of MMT and LPS (Figure 2A). LPS (100 ng/mL) induced TNFα and IL-1β mRNA expression in both sham and MMT-treated hPBMCs (Figure 2B). Under sham conditions, TNFα and IL-1β mRNA expression increased from 2 to 6 h. In contrast, MMT suppressed both TNFα and IL-1β mRNA expression at 2 h, which was maintained at 6 h; compared to sham+LPS, MMT reduced TNFα by 77% and IL-1β by 67%, though these differences did not reach statistical significance.

To further investigate the effect of MMT on the priming phase, we measured canonical NF-κB activity, the master regulator of inflammation.^11^ NF-κB activity was measured in THP-1 (human) and RAW264.7 (murine) cells stably expressing luciferase under the control of an NF-κB-responsive promoter. To evaluate the time-dependency of MMT immunomodulatory effects on LPS-induced responses in THP-1 and RAW264.7 cells, two approaches were employed: (i) co-treatment, in which cells were exposed to MMT and LPS simultaneously and (ii) pre-treatment, in which MMT was applied to cells 24 h prior to LPS stimulation (detailed in Figure 2Cii and iii). In both strategies (Figure 2D), MMT treatment markedly reduced NF-κB-driven luciferase activity in both THP-1 and RAW264.7 cells.

We next examined the LPS-induced NF-κB signaling pathway. Upon LPS stimulation, NF-κB translocates to the nucleus and activates transcription. Phosphorylation of p65 at Ser536 is essential for this activation and serves as a marker of NF-κB activity.^28^ Using immunofluorescence, we quantified p65 (Serine 536) phosphorylation in THP-1 cells 60 min after LPS stimulation (100 ng/mL). At this time point, LPS induced an 85% increase in p65 phosphorylation relative to vehicle control. Consistent with the observed reduction in NF-κB activity, MMT attenuated LPS-induced p65 (Ser536) phosphorylation by 34%, although this decrease did not reach statistical significance (Figure 2E).

### MMT suppressed LPS-sensitization to ATP-induced pyroptosis

We next analyzed pyroptosis, the inflammasome-activated form of programmed cell death characterized by membrane rupture and the release of mature pro-inflammatory IL-1β and IL-18 ^27^. THP-1 cells, which express NLRP3 and upregulate it in response to LPS,^14^ were used to determine sensitivity to extracellular ATP, a physiological damage-associated molecular pattern that activates inflammasomes.^27^

To measure the impact of MMT on LPS priming for pyroptosis, THP-1 cells were treated with vehicle (DMSO) or LPS (100 ng/mL) for 5 h and then additionally treated with ATP (5 mM) for 1 h. At 6 h, dead and damaged cells were detected by propidium iodide (PI) staining (Figures 2F and 2G). MMT had no effect on ATP-induced cell death in the absence of LPS priming, indicating that MMT does not impact basal inflammasome activation (Figure 2H). However, while LPS sensitized sham-treated THP-1 cells to ATP-induced pyroptosis, MMT significantly suppressed this sensitization by 26%, reducing cell death to levels comparable to ATP treatment alone.

Collectively, these *in vitro* findings demonstrate that MMT exerts anti-inflammatory effects on mononuclear immune cells when applied prophylactically (pre-treatment) or acutely (co-treatment). MMT suppresses NF-κB activity, thereby reducing pro-inflammatory cytokine production and LPS-sensitivity to ATP-activated pyroptosis.

### MMT reduced LPS-induced microgliosis and astrogliosis in rat brain

To evaluate the *in vivo* effects of MMT on glial activation and neuronal damage, we employed intracerebral LPS injection in rats, an established rodent model for neuroinflammation^29^^,^^30^^,^^31^ that induces *in vivo* activation and expansion of microglia (microgliosis) and astrocytes (astrogliosis).^32^ Injection into the substantia nigra (SN) provides a robust model for studying the progression of neuroinflammation in neurodegenerative diseases, enabling investigation of the interplay of gliosis, cytokine release, and ROS production.^33^^,^^34^

**Figure 3:**
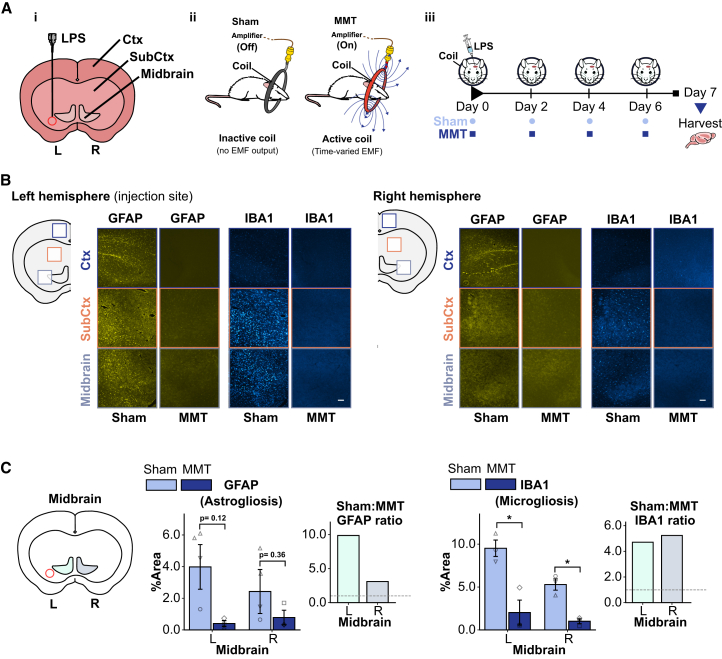
MMT reduced gliosis activation in a rat model of LPS-induced neuroinflammation (A) (i) Schematic of the midbrain showing the site of LPS injection, (ii) transcranial MMT exposure setup for rat and (iii) treatment protocol. *N* = 3 male rats per treatment group (Sham and MMT). See also Figures S1 and S2, and Table S1. (B) Representative immunofluorescence images of astrocytes (GFAP, yellow) and microglia (IBA1, blue) in left and right hemispheres, with regions of interest (ROIs) indicated: cortex (blue box), sub-cortex (orange box) and midbrain (gray box). ROI image (1,000 μm × 1,000 μm); scale bar, 100 μm. (C) Quantification of GFAP- and IBA1-positive areas in midbrain ROIs for both hemispheres. Bars show percent area occupied by astrocytes and microglia in sham- and MMT-treated groups. Left and right hemisphere ratios of GFAP- and IBA1-positive areas are shown for sham-to-MMT treatments, indicating the extent of pathology spread from the left (injected) to right hemisphere. Images were obtained using a 10× objective. Data are presented as mean ± SD. Statistical significance was determined by unpaired *t* test. ∗*p* < 0.05, ∗∗*p* < 0.01, ∗∗∗*p* < 0.001.

Rats received unilateral LPS injection into the left hemisphere midbrain, followed by transcranial MMT or sham treatment for 15 min every 48 h over 7 days (Figure 3A). Sham-treated rats were treated identically to the MMT group, except that the coil positioned over the head was inactive and generated no EMF output. On day 7, animals were sacrificed and brain sections were analyzed by immunohistochemistry to assess microglial and astrocytic responses. Sections were stained with anti-GFAP to detect astrocytes and anti-IBA1 to detect microglia (example regions shown in Figure 3B). Anti-tyrosine hydroxylase was used to identify the SN region for analysis. Deep learning-assisted segmentation^35^ was used to quantify the percent area occupied by GFAP- and IBA1-positive cells in regions of interest (ROIs), from cortical, subcortical, and midbrain regions of both hemispheres (Figure 3B). The workflow is described in STAR Methods and Figure S2 and Table S1.

Intermittent MMT treatment (15 min every 48 h) significantly suppressed gliosis compared to sham treatment, with the most pronounced effects in the midbrain. MMT reduced astrogliosis (GFAP-positive area) by 90% in the ipsilateral hemisphere and 68% in the contralateral hemisphere, and reduced microgliosis (IBA1-positive area) by 79% and 81%, respectively (Figure 3C). Left and right hemisphere ratios of GFAP- and IBA1-positive areas for sham-to-MMT treatments are shown for each region (Figures 3C and S1). Similar but less pronounced reductions were observed in the cortex and subcortex (Figures 3B and S1). Table S1 summarizes the % area values for GFAP and IBA1 immunoreactivity across all analyzed brain regions, showing region-specific effects of MMT relative to sham, with significant reductions in IBA1+ microglial area observed in the midbrain.

### MMT reduced LPS-induced tissue pathology and lesion size in rat brain

**Figure 4:**
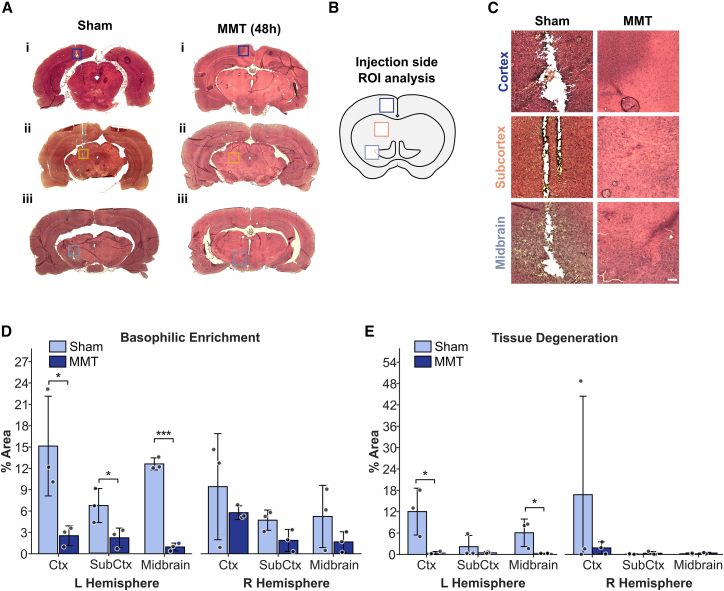
MMT reduced histopathological markers of neuroinflammation in LPS-injected rat brain (A) Representative H&E-stained coronal brain sections from sham- and MMT-treated rats following unilateral LPS injection (treatment protocol overview in Figure 3Ai and ii). Boxes indicate regions of interest (ROIs) for quantitative analysis. See also Figure 3 and Table S2. (B) Schematic illustrating cortical and sub-cortical regions within ROIs for cortex, subcortex and midbrain used to quantify basophilic enrichment (indicative of immune cell infiltration) and tissue degeneration (voids indicating cell loss). (C) Higher magnification of ROIs from (A) showing cortex, subcortex and midbrain in the injected left hemisphere of sham and MMT treated rats. ROI image (860 μm × 860 μm); scale bar, 100 μm. (D) Quantification of basophilic enrichment expressed as % area for ROIs in cortex (Ctx), subcortex (SubCtx), and midbrain of left and right hemispheres. (E) Quantification of tissue degeneration expressed as % area for ROIs in cortex, subcortex, and midbrain of left and right hemispheres. Images were obtained using a 10× objective. Data are presented as mean ± SD. Statistical significance was determined by unpaired *t* test. ∗*p* < 0.05, ∗∗*p* < 0.01, ∗∗∗*p* < 0.001.

Hematoxylin & eosin (H&E) staining revealed histopathological changes in the rat brain following LPS injection into the left hemisphere midbrain (Figure 4A). Sham-treated animals exhibited a pronounced pathology in both hemispheres, including dark basophilic staining, indicative of mononuclear cell infiltration, as well as prominent lesions reflecting tissue degradation and loss.^36^ Both features were also apparent in the noninjected right hemisphere. In contrast, MMT-treated rats exhibited minimal pathological changes in either hemisphere.

To quantify these features, ROIs from the cortical, subcortical and midbrain regions (Figures 4A–4C) were analyzed using Weka machine learning-assisted image analysis^37^ to measure the percent area occupied by basophilic regions (indicating immune cell infiltration) and tissue voids (indicating neurodegeneration). The image analysis workflow is shown in Figure S3.

Overall, sham-treated, LPS-injected rats exhibited markedly greater basophilic enrichment and tissue degeneration compared to LPS-injected, MMT-treated rats (Figures 4D and 4E; Table S2). In the injected (left) hemisphere, MMT significantly reduced basophilic infiltration by 83% in the cortex, 68% in the subcortex, and 95% in the midbrain (Figure 4D). Tissue degeneration followed a similar pattern of reduction, with decreases of 97% in the cortex, 84% in the subcortex, and 97% in the midbrain (Figure 4E and Table S2). Analysis of the combined ROI dataset for the injected left hemisphere highlights these effects, showing an 84% reduction in basophilic enrichment and a 94% reduction in tissue degeneration (Table S2). Although not statistically significant, a similar trend was observed in the non-injected (right) hemisphere, where MMT reduced basophilic enrichment by 52% and tissue degeneration by 85%.

Together, these results demonstrate that MMT significantly suppressed the LPS-induced neuroinflammatory response, characterized by microglial activation, peripheral immune cell infiltration, and neurodegeneration within cortical and subcortical regions. *In vivo*, MMT applied every 48 h effectively mitigated LPS-induced gliosis and tissue pathology, providing evidence that MMT limits glial reactivity and preserves structural integrity in response to a robust neuroinflammatory insult.

### MMT reduced neuro-immune interactions mediating neuronal oxidative stress

**Figure 5:**
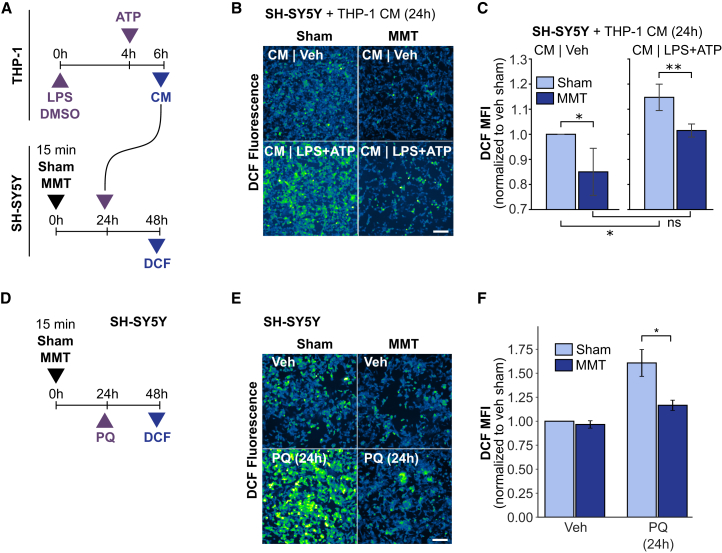
MMT reduced immune-mediated neuronal oxidative stress and enhanced neuronal antioxidant capacity (A) Schematic of *in vitro* neuroimmune interaction model. THP-1 cells were treated with vehicle (DMSO) or LPS (100 ng/mL) for 4 h followed by ATP (5 mM) for 2 h, and conditioned media (CM) was collected at 6 h. SH-SY5Y cells were exposed to sham or MMT, and then 24 h later treated with CM for an additional 24 h. Intracellular ROS levels were measured by H_2_DCF oxidation and fluorescence imaging. See also Figure S4. (B) Representative images of DCF fluorescence in sham- and MMT-treated SH-SY5Y neurons exposed to 24 h of THP-1 vehicle or LPS+ATP CM for 24 h (20× magnification; scale bars, 100 μm). (C) Quantification of DCF fluorescence in SH-SY5Y cells. (D) Schematic of direct neuronal oxidative stress model. SH-SY5Y cells were exposed to sham or MMT, then 24 h later treated with paraquat (PQ, 1 μM) for 24 h, followed by ROS detection by DCF fluorescence imaging. (E) Representative images for DCF fluorescence in sham- and MMT-treated SH-SY5Y neurons treated with PQ for 24 h (scale bars, 100 μm). (F) Quantification of DCF fluorescence in SH-SY5Y cells. Data are presented as mean ± SD. Statistical significance was determined by unpaired *t* test. ∗*p* < 0.05, ∗∗*p* < 0.01, ∗∗∗*p* < 0.001.

Finally, to gain insights into whether the observed *in vivo* MMT effects resulted from modulation of immune responses, and/or the activation of intrinsic neuronal protective mechanisms, we developed an *in vitro* model for immune cell-to-neuron signaling, using THP-1 monocyte conditioned media (CM) applied to SH-SY5Y neuronal cell lines (Figure 5A). To simulate an inflammation-induced neurotoxic environment, THP-1 monocytes were treated with DMSO (vehicle) conditions, or 4 h LPS (100 ng/mL) and an additional 2 h of ATP (5 mM), and subsequently CM was collected. To investigate neuronal effects, human neuroblastoma SH-SY5Y cells were treated with either sham or MMT, and 24 h post-treatment exposed to vehicle or LPS+ATP CM for 24 h. As oxidative stress has a critical role in the onset and progression of several brain disorders including neurodegenerative diseases,^38^^,^^39^ after 24 h of CM treatment, SH-SY5Y neurons were analyzed for levels of intracellular ROS production, measuring oxidation of H_2_DCF in populations of single cells. Notably, in SH-SY5Y cells treated with CM from vehicle-treated THP-1 cells (Figures 5B and 5C), MMT caused a small but significant decrease in DCF fluorescence. In contrast, CM from LPS+ATP-treated THP-1 cells markedly increased DCF fluorescence in sham-treated SH-SY5Y cells, whereas MMT treatment reduced DCF fluorescence by 22%, restoring ROS levels close to baseline.

### Neurotoxic complex I ROS production was reduced by MMT

In addition to dysregulated immune responses, neuronal oxidative stress characterizes neurodegenerative diseases and severe mental health disorders.^10^^,^^40^ To that end, we simulated a chronic state of oxidative stress through exposure to paraquat (PQ), a redox cycler^41^ which increases mitochondrial ROS production at complex I.^42^ PQ is used as a surrogate to investigate neuroinflammation and mechanisms of neurodegeneration in animal and cell culture models and exposure due to use as pesticide increases risk of Parkinson’s disease.^43^ At 24 h following exposure to MMT or sham, SH-SY5Y cells were treated with PQ (1 μM) for an additional 24 h, at which time ROS were quantified by DCF fluorescence (Figure 5D). In sham-treated SH-SY5Y cells, PQ resulted in a 52% increase in DCF fluorescence, indicating significantly higher levels of ROS production. In contrast, MMT-treated SH-SY5Y cells exhibited only a 20% increase, representing a significant attenuation of PQ-induced oxidative stress (Figures 5E and 5F). Figure 5 results suggest that *in vivo* MMT effects occur through both the modulation of the immune response and activation of an intrinsic neuroprotection mechanism.

## Discussion

**Figure 6:**
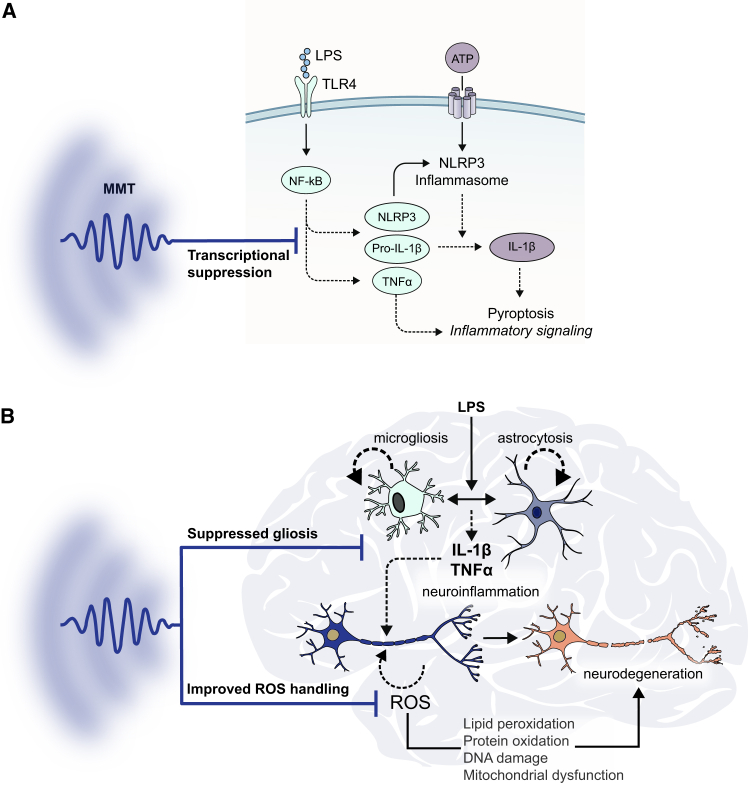
Mechanisms underlying anti-inflammatory and neuroprotective effects of MMT (A) Immune cell anti-inflammatory mechanisms. MMT suppresses NF-κB signaling, reducing transcription of pro-inflammatory cytokines (TNFα, IL-1β) and decreasing inflammasome priming, thereby attenuating sensitivity to ATP-induced pyroptosis. Dashed lines indicate pathways suppressed by MMT. (B) Neuronal protective mechanisms. MMT exerts neuroprotection through two complementary pathways: (1) indirect effects via suppression of glial activation and inflammatory cytokine production, and (2) direct effects through enhanced intrinsic neuronal antioxidant capacity and resistance to mitochondrial oxidative stress. Dashed lines indicate pathways modulated by MMT.

This study demonstrates that MMT, a locally administered, 15-min radiofrequency protocol, produces anti-inflammatory and neuroprotective effects across multiple experimental models. In immune cells, MMT suppressed LPS-induced NF-κB transcriptional activity, cytokine production, and pyroptotic cell death. In neurons, MMT reduced both cytokine-driven and toxin-induced oxidative stress, with protective effects persisting up to 48 h post MMT exposure. *In vivo*, noninvasive transcranial MMT mitigated glial activation and prevented neuronal loss in a rodent model of LPS-induced progressive neuroinflammation. Together, these findings provide evidence that MMT triggers durable cross-cellular reprogramming of inflammatory and oxidative stress responses rather than transient field effects. An overview of MMT effects is proposed in Figure 6.

The observed dual-compartment effects, NF-κB inhibition in immune cells and enhanced antioxidant capacity in neurons, suggest MMT engages redox-sensitive transcriptional networks, possibly with reciprocal crosstalk between NRF2 and NF-κB. NRF2, the master regulator of cellular antioxidant responses,^44^ is well understood to suppress inflammatory ROS production^45^ and inhibit NF-κB-mediated cytokine transcription through direct and indirect effects.^46^^,^^47^^,^^48^^,^^49^ Indeed, NRF2 has emerged as an early, sensitive target to both static magnetic fields (sMFs) and TV-EMF. EMF in the extremely low frequency range modulates NRF2 signaling in an exposure-dependent manner,^50^ and high-intensity repetitive magnetic stimulation results in NRF2 activation within minutes.^51^ In addition, long-term, multi-day exposures to sMFs^52^ and the combination of sMF and electric fields (sBE)^22^ activate NRF2 in mouse liver. Importantly, *in vivo* rodent studies have evidenced the potential of magnetic field NRF2 activation as a therapeutical approach, as NRF2 activation coincides with the reversal of insulin resistance following sMF and sBE treatments^22^^,^^52^ and improvements in depressive behavior in response to transcranial magnetic stimulation.^53^

Additional signaling modules may participate upstream of NRF2 activation. These include the suppression of p38 MAPK, which is upstream of LPS-induced NF-κB activity^54^ and or activation of SIRT1, which directly represses NF-κB signaling,^55^ both reported TV-EMF targets.^56^^,^^57^ In addition, TV-EMFs and sMFs modulate ROS production and mitochondrial function,^22^^,^^58^^,^^59^ which are upstream of NRF2 and NF-κB,^60^ and critical drivers of hormetic stress response programs involving NRF2,^22^ HIF1,^61^ and mitophagy^62^ pathways. To elucidate the MMT mechanism(s) of action, molecular level analyses are needed to determine whether these interactions explain how MMT restrains acute inflammatory responses while promoting cellular homeostasis.

Our findings that MMT robustly interfered with early immune activation signaling and promoted neuroprotective effects align with earlier studies, including TV-EMF reduction of IL-1β levels following TBI,^23^ suppressed pro-inflammatory cytokine secretion,^18^ attenuated joint inflammation and damage in a murine model of rheumatoid arthritis,^63^ reduced IL-1β and pain following breast reconstruction,^24^ and reduced oxidative stress in models of neurodegeneration.^61^^,^^64^ Going forward, exploring how different exposure parameters (frequency, intensity, and exposure duration) differentially engage EMF-sensitive pathways will drive the design of optimized protocols for specific therapeutic applications. This parameter-response relationship is critical, as variations in EMF parameters can shift responses from anti-inflammatory to pro-inflammatory.^65^^,^^66^ Defining these therapeutic ranges will be essential for translating TV-EMF approaches into reliable clinical interventions.

### Limitations of the study

While this study demonstrates reproducible anti-inflammatory and antioxidant effects of MMT across cellular and *in vivo* models, several limitations should be noted. Direct *in vivo* detection of ROS was not performed, and redox modulation is inferred from cell model and histological outcomes. Further elucidation of MMT-targeted signaling, including the relative roles of NF-κB, NRF2, and related pathways, will require pharmacological or genetic perturbation studies, and *in vivo* verification of redox modulation. In addition, our *in vitro* analyses relied on THP-1 CM applied to SH-SY5Y cells. While this approach captured novel cytoprotective MMT effects on immune-neuronal interactions, future studies incorporating microglial models and more physiologically relevant neuronal systems, such as primary neurons, iPSC-derived neuronal subtypes, or organoids, will be essential to more accurately model MMT effects on brain-resident immune-neuronal dynamics. Finally, although this study demonstrated beneficial effects of transcranial MMT with repeated dosing, longer treatment paradigms and disease-specific models will be needed to assess durability and therapeutic relevance.

In conclusion, MMT represents a novel biophysics-based approach to address underlying pathological components of neuroinflammatory disorders and opens new possibilities for non-invasive intervention beyond traditional pharmacological and neuromodulation approaches. The sustained cellular state changes we observed following single MMT exposures, and cumulative neuroprotective effects in a rodent model of progressive neuroinflammation, with repeated dosing, position MMT as a candidate intervention for diseases characterized by chronic neuroinflammation, oxidative stress, and progressive neuronal loss, including neurodegenerative diseases, neurodevelopmental disorders, and severe mental health disorders.^4^

Our multidisciplinary program spans molecular, cellular, and *in vivo* models, demonstrating MMT efficacy from gene-level modulation to tissue-level neuroprotection in rodents, and now extends to a clinical study in post-acute sequelae of SARS-CoV-2,^67^ aligning with recent pharmacological efforts to mitigate virus-related neuroinflammation.^68^^,^^69^ Together, these preclinical and clinical efforts establish a reciprocal translational framework in which mechanistic insights from cellular and animal models inform the design of human treatment protocols, while clinical findings feedback to advance understanding of mechanistic models. This bench-to-bedside-to-bench reciprocity represents a departure from traditional drug development paradigms and provides a compelling proof-of-concept for biophysical interventions such as MMT as emerging therapeutic modalities for neurological and psychiatric disorders.

## Resource availability

### Lead contact

Further information and requests for resources and reagents should be directed to and will be fulfilled by the lead contact, Blake T. Gurfein (b@fareon.co).

### Materials availability

This study did not generate new unique reagents.

### Data and code availability

All data generated or analyzed during this study are included in this published article and its supplemental information files. All data reported in this paper will be shared by the lead contact upon request.

This paper does not report original code. Custom image analysis workflows used to support the findings of this study are available from the lead contact upon request.

Any additional information required to reanalyze the data reported in this paper is available from the lead contact upon request.

## Acknowledgments

The authors thank the Gladstone Histology and Light Microscopy Core for assistance with histology and microscopy. This work was supported by Fareon, Inc.

## Author contributions

Conceptualization, G.A.T., K.M.T., and B.T.G.; methodology, N.N., N.R.B., G.A.T., K.M.T., and B.T.G.; investigation, N.N., N.R.B., and B.T.G.; data curation, N.N., N.R.B., and B.T.G.; formal analysis, N.N. and N.R.B.; writing – original draft, N.R.B.; writing – review & editing, N.N., N.R.B., G.A.T., K.M.T., and B.T.G.; visualization, N.R.B.; project administration, N.R.B. and B.T.G.; resources, G.A.T., K.M.T., and B.T.G.; supervision, B.T.G.; funding acquisition, B.T.G.

## Declaration of interests

The authors are employees and/or equity holders of Fareon, Inc. B.T.G. is an inventor on a patent application that is based in part on the work reported in this manuscript. No additional financial or personal conflicts of interest are declared. All other authors declare no competing interests.

## Declaration of generative AI and AI-assisted technologies in the writing process

During the preparation of this work, the authors used ChatGPT to assist in the generation of STAR Methods. After using this tool, the authors reviewed and edited the content as needed and take full responsibility for the content of the publication.

## STAR★Methods

### Key resources table

**Table:** 

REAGENT or RESOURCE	SOURCE	IDENTIFIER
**Antibodies**		

Rabbit polyclonal anti-Tyrosine Hydroxylase	MilliporeSigma	Cat# AB152; RRID: AB_390204
Goat polyclonal anti-IBA1	Invitrogen	Cat# PA5-18039; RRID: AB_10982846
Chicken polyclonal anti-GFAP	MilliporeSigma	Cat# ab5541; RRID: AB_177521
Rabbit monoclonal anti-phospho-NF-κB p65 (Ser536)	R&D Systems	Cat# MAB72261; RRID:N/A
Donkey anti-rabbit Alexa Fluor 488	Thermo Fisher Scientific	Cat# A-21206; RRID: AB_2535792
Donkey anti-goat Alexa Fluor 680	Thermo Fisher Scientific	Cat# A-21084; RRID: AB_2535741
Donkey anti-chicken Alexa Fluor 568	Thermo Fisher Scientific	Cat# A-78950; RRID: AB_2921072
CoraLite Plus 488 Goat Anti-Rabbit IgG	Proteintech	Cat# RGAR002; RRID: AB_3073506

**Chemicals, peptides, and recombinant proteins**		

ATP, sodium salt	InvivoGen	Cat# tlrl-atpl
Paraquat dichloride hydrate	Sigma-Aldrich	Cat# 36541
H_2_DCFDA (2',7'-dichlorodihydrofluorescein diacetate)	AAT Bioquest	Cat# 15204
Propidium iodide	Invitrogen	Cat# P3566
D-Luciferin potassium salt	Gold Biotechnology	Cat# LUCNA-100
TRIzol Reagent	Invitrogen	Cat# 15596026
SuperScript III Reverse Transcriptase	Invitrogen	Cat# 18080044
Fast SYBR Green Master Mix	Applied Biosystems	Cat# 4385616
Hoechst 33342	Invitrogen	Cat# H3570
ProLong Gold Antifade Mountant (no DAPI)	Thermo Fisher Scientific	Cat# P36930
Escherichia coli O55:B5 Lipopolysaccharide (LPS)	Sigma-Aldrich	Cat# L2880
Escherichia coli O111:B4 Lipopolysaccharide (LPS)	Sigma-Aldrich	Cat# L3024

**Critical commercial assays**		

Human IL-1β Uncoated ELISA Kit	Invitrogen	Cat# 88-7261-88; RRID:AB_2575054
Human TNFα Uncoated ELISA Kit	Invitrogen	Cat# 88-7346-88; RRID: AB_2575097
BD Vacutainer CPT tubes	BD Biosciences	Cat# 362753

**Experimental models: Cell lines**		

RAW 264.7 (mouse macrophage, male)	ATCC	Cat# TIB-71; RRID: CVCL_0493
THP-1 (human monocytic, male)	ATCC	Cat# TIB-202; RRID: CVCL_0006
SH-SY5Y (human neuroblastoma, female)	AddexBio	Cat# C0005004; RRID: CVCL_0019
RAW 264.7 NF-κB reporter	G Timblin^70^	N/A
THP-1 NF-κB reporter	G Timblin^71^	N/A

**Oligonucleotides**		

Human Hprt Forward: CATTATGCTGAGGATTTGGAAAGG	Sigma-Aldrich	Custom oligo
Human *Hprt* Reverse: CTTGAGCACACAGAGGGCTACA	Sigma-Aldrich	Custom oligo
Human *Tnfα* Forward: CTCTTCTGCCTGCTGCACTTTG	Sigma-Aldrich	Custom oligo
Human *Tnfα* Reverse: ATGGGCTACAGGCTTGTCACTC	Sigma-Aldrich	Custom oligo
Human *Il-1β* Forward: CCACAGACCTTCCAGGAGAATG	Sigma-Aldrich	Custom oligo
Human *I-1β* Reverse: GTGCAGTTCAGTGATCGTACAGG	Sigma-Aldrich	Custom oligo

**Software and algorithms**		

Fiji (ImageJ)	https://imagej.net/software/fiji	RRID: SCR_002285
Napari v0.6	https://napari.org	RRID: SCR_022765
CellPose (Cyto3 model)	https://cellpose.org	RRID: SCR_021716
Trainable Weka Segmentation v3.3.2	https://imagej.net/plugins/tws	RRID: SCR_001214
Python 3.10	https://www.python.org	RRID: SCR_008394
Inkscape v1.4	https://inkscape.org	RRID: SCR_014479

**Other**		

BioTek Cytation 5 multi- mode reader	Agilent BioTek Instruments	Model: Cytation 5; RRID:SCR_019732
EVOS M7000 imaging system	Thermo Fisher Scientific	Model: AMF7000; RRID:SCR_025070
Operetta CLS High-content imaging System	Revvity	Model: CLS; RRID:SCR_018810
Nikon Eclipse Ti2 fluorescence microscope	Nikon Instruments	Model: Ti2; RRID:SCR_021068
Applied Biosystems QuantStudio 6	Thermo Fisher Scientific	Cat# 4485691; RRID:SCR_020239
GloMax Discover microplate reader	Promega	Model: GM3000; RRID: SCR_018614 (delete SCR_023469
Siglent SDS1202X-E oscilloscope	Siglent Technologies	Model: SDS1202X-E
Beehive Electronics near-field probe	Beehive Electronics	Model: 100A
Siglent SDG1062X signal generator	Siglent Technologies	Model: SDG1062X
NanoDrop spectrophotometer	Thermo Fisher Scientific	Model: NanoDrop 2000; RRID:SCR_018042
MMT EMF system	Fareon, Inc	Custom; 27.12 MHz

### Experimental model and study participant details

#### Human peripheral blood mononuclear cells (hPBMCs)

Fresh human peripheral blood was obtained from healthy adult donors through HumanCells BioSciences (Sunnyvale, CA, USA). Each donor was confirmed negative for HIV-1/2, HBV, HCV, and syphilis before collection**.** Samples were maintained and shipped at room temperature (20–25 °C) on the day of collection. Donor sex and age were disclosed by the vendor and recorded. A total of 11 independent donors were used for the PBMC ELISA experiments, and 2 independent donors were used for the qPCR experiments, as indicated in the figure legends. Each donor sample was processed individually and allocated to experimental groups based on the assay design (e.g., treatment versus control). No randomization or stratification by sex or age was applied. While samples from both male and female donors were included, the influence of sex on the results was not addressed. All donors provided written informed consent under Institutional Review Board (IRB)**-**approved protocols.

For PBMC isolation, whole blood was processed using BD Vacutainer® CPT™ tubes (BD Biosciences, Cat# 14-959-51D). Tubes were centrifuged at 1,700 × g for 25 minutes at 22 °C, plasma layers transferred, and cells washed twice with phosphate-buffered saline (PBS). Final cell pellets were resuspended in RPMI-1640 (Gibco 11875-093) supplemented with 10% FBS (Cytiva SH3091003HI). Viability was confirmed by trypan blue exclusion, and cells were adjusted to 2.5 × 10^5^ cells/mL before treatment.

#### Immortalized cell lines

The following cell lines were used: RAW 264.7 (murine macrophage; ATCC TIB-71; male origin), THP-1 (human monocytic; ATCC TIB-202; male origin), and SH-SY5Y (human neuroblastoma; AddexBio C0005004; female origin). Cell lines were obtained from verified sources and were not independently authenticated by our laboratory. All cell lines were routinely screened for mycoplasma using Hoechst 33342 nuclear staining. Cultures were examined for the presence of extranuclear fluorescent puncta indicative of mycoplasma DNA. No mycoplasma contamination was observed during the study period.

Culture conditions: RAW 264.7 and THP-1 cells were maintained in RPMI-1640 (Gibco 11875-093) supplemented with 10 % (v/v) fetal bovine serum (FBS) and 1 % penicillin–streptomycin (Gibco 15-140-122). SH-SY5Y cells were maintained in DMEM/F-12 (Gibco 11-320-033) with identical supplements. Cells were incubated at 37 °C, 5 % CO_2_, and 95 % relative humidity in a humidified atmosphere. All cultures were routinely monitored for morphology, confluence, and contamination according to quality control standards.

#### Rodent model of neuroinflammation

Experiments were performed under contract at Comparative Biosciences, Inc. (Sunnyvale, CA, USA; Study No. CB23-7021-R-EF). All procedures complied with the ARRIVE guidelines and were approved by the Comparative Biosciences Institutional Animal Care and Use Committee (IACUC).

##### Animals

Adult male Sprague–Dawley rats (Rattus norvegicus, 8–10 weeks of age; ∼300 g; Charles River Laboratories) were used. Rats were individually housed in static micro-isolator cages under a 12 hour light/dark cycle (lights on 0700–1900 h) at 20–26 °C and 30–70 % humidity, with Purina RAT LabDiet 5001 rodent chow and municipal tap water *ad libitum*. Temperature, humidity, and light cycle were logged daily. Animals were acclimated ≥3 days and trained in restraint procedures for 7 days before study start.

##### Experimental design

Rats were randomly assigned to two groups (Sham n = 3, MMT n = 3). Neuroinflammation was induced on Day 0 by unilateral stereotactic injection of lipopolysaccharide (LPS, E. coli O111:B4; Sigma-Aldrich L3024, 1 mg/mL in 0.9 % NaCl) into the left substantia nigra (5.5 mm posterior, 1.8 mm lateral, 8.3 mm ventral to bregma; Paxinos & Watson atlas).

##### Surgical and peri-operative procedures

Anesthesia was maintained with isoflurane. LPS (2 μL) was injected slowly into the target site using a stereotactic syringe pump. Following injection, wounds were sutured, and animals received standard postoperative care including buprenorphine SR (1.0–1.2 mg/kg subcutaneous (s.c.)), enrofloxacin (5 mg/kg s.c.), and lactated Ringer’s solution (5 mL s.c.). Recovery was monitored until full mobility was regained.

##### MMT treatment

Beginning immediately after surgery, animals received either active MMT or sham exposure for 15 minutes once every 48 hours over 7 days. The active MMT treatment delivered a time-varied electromagnetic field (EMF) at 27.12 MHz using a loop inductive coil. Sham-treated animals were placed in identical restraint and coil configurations, but no current waveform was passed through the coil during exposure (Figure 3ii).

##### Clinical observations

Animals were observed daily for activity, behavior, and signs of discomfort. Body weight and food intake were recorded at baseline and on Day 7. No unanticipated adverse effects were observed.

##### Tissue collection

On Day 7, animals were anesthetized with isoflurane and perfused transcardially with heparinized saline followed by 4 % paraformaldehyde (PFA). Brains were weighed, rinsed in PBS (4 °C), and cryoprotected in serial 10 %, 20 %, 30 % sucrose solutions (1:20 tissue:solution ratio) at 4 °C. Fixed tissues were transferred to the Gladstone Institute Histology Core (San Francisco, CA) for sectioning and immunohistochemical analysis.

### Method details

All experimental procedures were designed to evaluate the effects of MMT on neuroinflammatory and oxidative stress responses *in vitro* and *in vivo*. The following sections describe in detail the protocols for electromagnetic exposure, LPS stimulation, histology, image acquisition, and quantitative analysis.

#### MMT exposure system and verification

All electromagnetic field exposures were delivered using a custom-built MMT system developed and operated by Fareon, Inc. (San Francisco, CA, USA) (Figure 1Ai). The setup consisted of a custom loop inductive coil, custom broadband RF amplifier, and Siglent 1062x signal generator producing a time-varied carrier frequency of 27.12 MHz (industrial, scientific, and medical band). The coil geometry and amplifier output were tuned to yield a magnetic flux density of approximately at the coil center. Field strength and waveform integrity were verified using a Beehive Electronics near-field probe coupled to a Siglent SDS1202X-E digital oscilloscope.

For *in vivo* experiments, the coil was positioned directly above the rat skull, centered over the injected hemisphere, as described above.

For *in vitro* assays, hPBMCs were exposed to sham or active MMT conditions at a density of 2.5 × 10^5^ cells/mL, and immortalized cell lines (RAW 264.7, THP-1, and SH-SY5Y) at 1 × 10^6^ cells/mL. Exposures were conducted in 6-well tissue culture plates using the same loop inductive coil and field parameters as described above (time-varied 27.12 MHz carrier frequency, 15-minute duration).

For sham conditions, cell culture plates were positioned on the laboratory bench adjacent to the active coil, beyond the range of detectable magnetic flux or induced electric field, as verified by field mapping with a near-field probe (Figure 1Aii). Ambient field measurements confirmed that residual EMF levels at the sham position were indistinguishable from background.

#### Immunofluorescence staining of tissues

Coronal brain sections were air-dried for 1 hour at room temperature (RT), fixed in cold methanol for 30 minutes at −20 °C, and briefly air-dried again. After two PBS washes (5 minutes each), sections were outlined with a PAP pen, rinsed in PBST (0.05 % Tween-20 in PBS), and blocked for 1 hour at RT in universal blocking buffer containing 0.1 % Triton X-100. Without additional washing, sections were incubated overnight at 4 °C with primary antibodies diluted in blocking buffer: rabbit anti-tyrosine hydroxylase (MilliporeSigma, Cat# AB152), goat anti-IBA1 (Invitrogen, Cat# PA5-18039), and chicken anti-GFAP (MilliporeSigma, Cat# ab5541). Negative controls received antibody diluent only. After equilibration to RT (30 minutes) and PBST washes (2 × 5 min, 1 × 10 min), sections were incubated for 30 minutes in the dark with donkey anti-rabbit Alexa 488 (Thermo Fisher, Cat# A21206), donkey anti-goat Alexa 680 (Thermo Fisher, Cat# A21084), and donkey anti-chicken Alexa 568 (Thermo Fisher, Cat# A78950). Sections were washed three times in PBST (5 minutes each), counterstained with DAPI (1 μg/mL, 10 minutes), washed, transferred to PBS, and mounted with ProLong Gold Antifade Mountant (no DAPI; Thermo Fisher, Cat# P36930). Stained slides were stored at 4 °C in the dark until imaging.

#### Hematoxylin and eosin (H&E) staining

Fixed brain tissue was dehydrated, embedded in paraffin, and sectioned using a rotary microtome (5 μm thickness). Sections were incubated at 60 °C for 30 minutes, deparaffinized in xylene (3 × 3 minutes), and rehydrated through graded ethanol (100 %, 95 %, 70 %, 2 minutes each) to distilled water. Slides were stained with Harris hematoxylin (4 minutes), rinsed in running tap water, blued in 0.2 % ammonia water (2 minutes), and counterstained with eosin Y (1 minute) after passage through 70 % ethanol. Dehydration was completed with 95 % and 100 % ethanol (2 × 2 minutes) followed by xylene (2 × 2 minutes), and sections were coverslipped using xylene-compatible mounting medium. Stained slides were dried and stored at room temperature until imaging.

#### Microscopy

Brightfield imaging of H&E-stained sections was performed at 10× magnification using an EVOS M7000 Imaging System (Thermo Fisher Scientific). Fluorescence imaging of immunostained brain sections was performed at 10× magnification on a Nikon fluorescence microscope equipped with standard FITC, TRITC, and Cy5 filter sets.

Fluorescence imaging of ROS and cell-death assays was carried out at room temperature using a Cytation 5 Cell Imaging Multi-Mode Reader (Agilent BioTek Instruments). For propidium iodide (PI) detection, cells were imaged at 10× magnification in black polystyrene 96-well plates (Costar, Corning, Cat# CLS3603). For DCF fluorescence measurements, cells were imaged at 20× magnification in 96-well glass-bottom plates (#1.5 high-performance coverglass; Cellvis, Cat# P96-1.5H-N). For phospho-p65 detection, cells were imaged at 10× magnification in 96-well glass-bottom plates using the EVOS M7000 imaging system. For high-resolution imaging of THP-1 cells during pyroptosis, sham and MMT-treated THP-1 cells were seeded in Cellvis 96-well plates and primed 5 hours with LPS (100 ng/mL), followed by 1 hour with the addition of ATP (5 mM). Cells were stained with Hoechst 33342 to label all nuclei, and PI to detect cells with compromised membrane integrity. Z-stacks of Hoechst, PI and brightfield were acquired with a 63× water immersion objective (NA 1.2) at 0.3 μm step intervals, and maximum-intensity projections were generated using ImageJ/Fiji. All image acquisitions were performed with identical exposure times, illumination intensity, and gain settings within each experiment.

#### Image analysis

##### Quantification of brain section gliosis

Immunofluorescence (IHC) images were processed and analyzed in Napari (v0.6) using the CellPose Counter plugin with the oneclick_Cyto3 enhancement model for initial cell detection.^72^ Images labeled for GFAP and IBA1 were then segmented using the Convpaint segmentation tool^35^ to identify specifically stained astrocytes and microglia. Regions of interest (ROIs) corresponding to cortical, subcortical, and midbrain areas were manually annotated to define GFAP-positive astrocytes and IBA1-positive microglia. These annotations were used to train Convpaint to segment the respective glial populations across entire tissue sections. Segmented binary masks were quantified in Fiji (ImageJ2 v2.16) to calculate the percent area occupied by GFAP- and IBA1-positive regions. The complete analysis workflow is illustrated in Figure S2.

##### Quantification of brain section histopathology

For H&E-stained sections, segmentation was performed using the Trainable Weka Segmentation plugin in Fiji to classify basophilic (darkly stained) and void (tissue-damaged) regions. ROIs corresponding to cortical, subcortical, and midbrain regions were manually annotated based on three morphological features: (1) pink cytoplasmic regions, (2) dark purple nucleic acid-dense regions indicating immune infiltration, and (3) pale or unstained regions representing tissue deterioration. These annotations were used to train the model to recognize comparable histopathological features across entire sections. Segmented binary images were quantified in Fiji to determine the percent area of basophilic and void regions. The analysis workflow is shown in Figure S3.

##### Cell line analysis

For SH-SY5Y DCF ROS assays and NF-κB immunofluorescence, cells were automatically segmented using CellPose (Cyto3 model) in Napari, and mean fluorescence intensity was measured per cell using scikit-image (v0.2) in Python (v3.10). The DCF and NF-κB analysis pipelines are shown in Figure S4. For THP-1 cell-death assays, the Napari CellPose Counter plugin was used to quantify total cell counts, and the Fiji Find Maxima plugin was applied to identify propidium iodide-positive cells. Fiji was used to assemble representative tissue and cell line images as well as data plots, and final figures were prepared using Inkscape v1.4.

#### Inflammatory stimulation and MMT treatment

To model neuroinflammatory activation, hPBMCs, THP-1, and RAW 264.7 cells were stimulated with lipopolysaccharide (LPS) derived from Escherichia coli O55:B5 (Sigma-Aldrich, Cat# L2880) or O111:B4 (Sigma-Aldrich, Cat# L3024) at final concentrations ranging from 10 ng/mL to 1 μg/mL in culture medium. Cells were incubated with LPS for 2–24 h, depending on the experimental design.

When indicated, adenosine triphosphate (ATP; 5 mM, InvivoGen, Cat# tlrl-atpl) was added for the final 1 h to promote NLRP3 inflammasome activation and pyroptotic signaling. Vehicle controls received equivalent volumes of PBS or DMSO.

Cells were exposed to active MMT or sham conditions either immediately before or concurrent with LPS stimulation, following the parameters described under MMT exposure system and field calibration (time-varied 27.12 MHz, 15-minute exposure). For combinatorial treatments, MMT exposure was applied either prior to or following LPS ± ATP stimulation, as indicated in figure schematics and legends.

After treatment, supernatants were collected for cytokine analysis, and cells were processed for NF-κB activation, ROS quantification, and viability assays. Pyroptotic cell death was assessed by propidium iodide uptake (Invitrogen, Cat# P3566).

#### Cytokine quantification by ELISA

Secreted cytokine levels were quantified from hPBMC culture supernatants following LPS ± MMT treatments. Samples were clarified by centrifugation (300 × g, 5 min, 22 °C) and analyzed immediately or stored at –20°C until use.

Human IL-1β and TNFα were measured using uncoated HRP-based ELISA kits following the manufacturer’s protocols: IL-1β: Human IL-1β Uncoated ELISA Kit (Invitrogen, Cat# 50-112-9046) and TNFα: Human TNFα Uncoated ELISA Kit (Invitrogen, Cat# 50-112-9050).

Supernatants were diluted appropriately in the assay buffer, added to pre-coated plates with capture antibody, and incubated for 2 h at room temperature. After washing, biotin-conjugated detection antibody, streptavidin-HRP, and TMB substrate were applied sequentially. Absorbance was measured at 450 nm using a BioTek Cytation 5 microplate reader, and cytokine concentrations were calculated from standard curves generated using recombinant protein standards provided in each kit.

All assays were performed in technical duplicates, and mean values were used for subsequent analysis.

#### Quantitative PCR (qPCR)

Total RNA was extracted using TRIzol Reagent (Invitrogen, Cat# 15-596-026) following the manufacturer’s protocol. RNA concentration and purity were verified by spectrophotometry (A_260_/A_280_ ≥ 1.8). One microgram of total RNA was reverse-transcribed into cDNA using SuperScript III Reverse Transcriptase (Invitrogen, Cat#18080-044) according to the manufacturer’s instructions.

Quantitative PCR was performed with Fast SYBR Green Master Mix (Applied Biosystems, Cat# 4385616) on an Applied Biosystems QuantStudio 6 real-time PCR instrument. Primers were synthesized by Sigma-Aldrich (custom oligos; sequences listed in key resources table). Each reaction (10 μL total volume) contained 1× SYBR mix, 0.3 μM of each primer, and 2 0 ng cDNA template. Thermocycling conditions were: 95 °C for 2 min, followed by 40 cycles of 95 °C for 15 s and 60 °C for 30 s. Melt-curve analysis confirmed amplification specificity.

Relative *Tnfα* and *Il-1β* mRNA expression was calculated using the ΔΔCt method, normalizing to *Hprt* as the internal reference gene.

#### NF-κB reporter assays

RAW264.7^70^ and THP-1^71^ cells stably expressing an NF-κB-responsive firefly luciferase reporter were used to quantify pathway activation. Cells were seeded at 1 × 10^6^ cells/mL in 6-well plates and subjected to sham or MMT conditions, followed by LPS stimulation (10-100 ng/mL, 4 h), as described in the results section. Following treatment, cells were transferred to white opaque 96-well plates (Corning, flat-bottom) for optimized luminescence detection. D-luciferin (Gold Biotechnology, Cat# LUCNA100) was added directly to the culture medium at a final concentration of 150 μg/mL, and luminescence was measured immediately using a Glowmax Discover plate reader. Each condition was tested in triplicate, and data were expressed as relative light units (RLU) normalized to vehicle control sham wells.

#### NF-κB phosphorylation assay

Phosphorylation NF-κB p65 at serine 536 was analyzed by immunofluorescence. At the indicated time points, cells were harvested by centrifugation (500 × g, 3 minutes, 4 °C) and immediately fixed with 4 % paraformaldehyde (10 min, RT) (Thermo Scientific, AAJ61899AK). After two PBS washes, cells were permeabilized using Permeabilization Buffer (eBioscience, Cat# 88-8824-00) for 10 minutes and blocked in PBS containing 5 % FBS for 1 hour at RT. Samples were incubated overnight at 4 °C with rabbit anti-phospho-NF-κB p65 (Ser536) (R&D Systems, Cat# MAB72261), diluted in PBS with 1% FBS, followed by CoraLite Plus 488 goat anti-rabbit IgG (Proteintech, Cat# RGAR002) and Hoechst 33342 nuclear stain (Invitrogen, Cat# H3570) for 1 hour at RT, washed twice with PBS, and imaged on an EVOS 7000.

#### Neuroimmune conditioned-media assay

To assess the paracrine effects of immune activation on neuronal oxidative stress, conditioned media (CM) were collected from THP-1 cell cultures following LPS + ATP ± MMT treatment, or treatment with vehicle control. After stimulation, cells and supernatants were harvested and centrifuged (500 × g, 5 minutes, 22 °C) to remove debris. The supernatants were transferred to new tubes and stored at –20°C until use.

Intracellular ROS was quantified using 10 μM H_2_DCF-DA (AAT Bioquest, Cat# 15204). Cells were incubated with H_2_DCF-DA for 1 hour at 37 °C in full medium, then washed twice with pre-warmed phenol red–free imaging medium before imaging. Fluorescence was measured using a BioTek Cytation 5 imaging reader (excitation 485 nm, emission 530 nm). Single-cell fluorescence intensities were quantified using Cellpose (Cyto3 model) in Napari, and values were normalized to sham-treated controls.

#### Paraquat-induced oxidative stress

To model mitochondrial reactive oxygen species (ROS) generation in neuronal cells, SH-SY5Y cells were treated with paraquat dichloride hydrate (Sigma-Aldrich, Cat# 36541). Cells were first exposed to sham or MMT conditions in 6-well plates, then immediately reseeded into 96-well plates at a density of 3 × 10^5^ cells per well in 200 μL of complete medium and allowed to attach overnight.

Intracellular ROS was quantified using 10 μM H_2_DCF-DA (AAT Bioquest, Cat# 15204). Fluorescence was measured using a BioTek Cytation 5 imaging reader (excitation 485 nm, emission 530 nm). Single-cell fluorescence intensities were quantified using Cellpose (Cyto3 model) in Napari, and values were normalized to sham-treated controls.

### Quantification and statistical analysis

All experiments were performed independently at least three times, unless otherwise indicated. Data are presented as mean ± standard deviation (SD). For imaging experiments, fluorescence intensity was normalized to sham control values. Statistical analyses were conducted in Python using the SciPy and Matplotlib libraries.

Comparisons between two groups were performed using two-tailed unpaired Student’s t-tests; paired t-tests were used for left-versus-right hemisphere comparisons. Statistical significance was set at p ≤ 0.05 and is denoted in figures as ∗ p ≤ 0.05, ∗∗ p ≤ 0.01, ∗∗∗ p ≤ 0.001. For notable non-significant trends, exact p values are reported.

Biological replicates were defined as independent donors for hPBMC and cell line experiments, and individual animals for rodent studies. Technical replicates within an experiment were averaged before analysis. Analysts performing ROI segmentation and quantification were blinded to treatment conditions. Sample sizes were informed by prior studies demonstrating similar effect sizes for LPS-induced neuroinflammation; formal power calculations were not performed.

### Additional resources

#### Description

Ongoing clinical trial evaluating at-home MMT treatment in post-acute sequelae of SARS-CoV-2 (PASC)-related cognitive dysfunction (ClinicalTrials.gov Identifier: NCT06739668; Humanity Neurotech [subsequently renamed Fareon, Inc.], registered January 2024).
