# Supplementary material for: Transcranial microtesla magnetic fields suppress neuroinflammation and neuronal oxidative stress burden
Source: iScience. 2025 Dec 15;29(1):114425. doi: 10.1016/j.isci.2025.114425 (PMC12803945; doi:10.1016/j.isci.2025.114425)
Supplement: Document S1. Figures S1–S4 and Tables S1 and S2 [file mmc1.pdf]

iScience, Volume *29*

Supplemental information

Transcranial microtesla magnetic fields suppress neuroinflammation and neuronal oxidative stress burden

Nhu Nguyen, Nathan R. Brady, Greg A. Timblin, Kevin M. Tharp, and Blake T. Gurfein

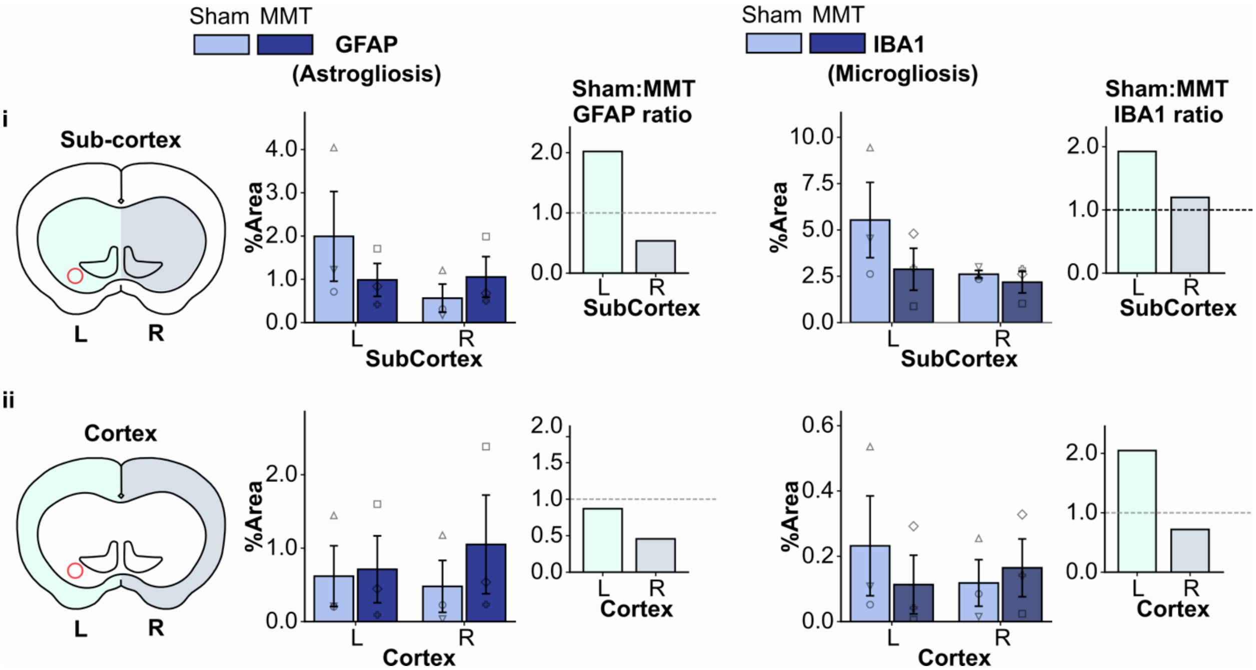

Supplemental Figure 1 (related to Figure 2C and Figure S2). Regional analysis of MMT effects on gliosis in cortex and subcortex.

Brain sections immunostained for GFAP and IBA1 were imaged at 10× magnification. Cortical, subcortical, and midbrain ROIs were defined, and representative $GFAP^{+}$ and IBA1+ areas were manually annotated to train the Convpaint segmentation algorithm. The trained model was then applied for automated segmentation across entire sections, and binary masks were quantified in Fiji to calculate the percent area occupied by GFAP+ and IBA1+ cells in the sub-cortex (i) and cortex (ii).

Data are presented as mean ± SD. Statistical significance was determined by unpaired t-test. * $p < 0.05$, ** $p < 0.01$, *** $p < 0.001$.

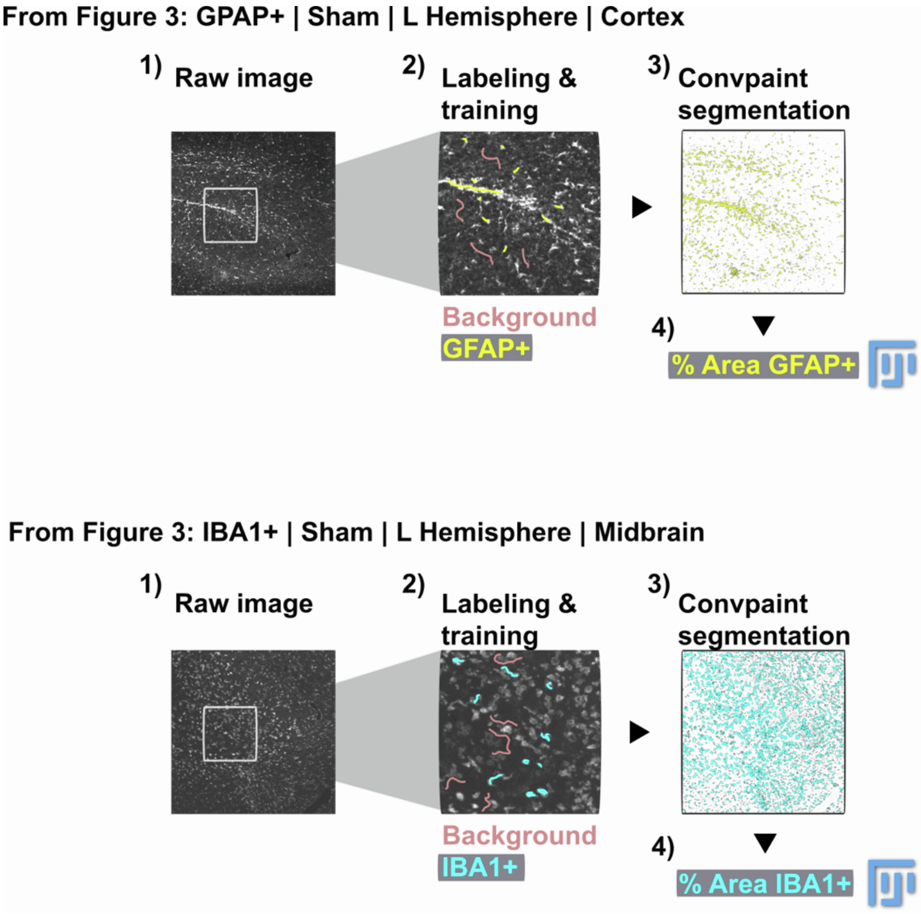

Supplemental Figure 2 (related to Figure 3). Convpaint-based segmentation workflow for quantifying GFAP+ and IBA1+ gliosis in brain sections.

Representative machine learning–assisted image analysis of immunofluorescence-stained brain regions following LPS injection. Two parallel workflows are shown for the cortex (top) and midbrain (bottom). GFAP+ %area quantification in yellow (top), and IBA1+ %area in cyan (bottom).

1. **Image acquisition**: Grayscale immunofluorescence micrographs of brain tissue were acquired at 10× magnification, cellular structures are shown as bright regions.
2. **Manual annotation and training**: Selected regions were manually annotated to distinguish target cell populations (GFAP+ astrocytes in yellow, IBA1+ microglia in

cyan) from background (red/brown). These annotations trained the Convpaint segmentation algorithm.

3. **Automated segmentation:** The trained model identified and segmented GFAP+ and IBA1+ cells across the entire raw image. Segmented areas show as color-coded (yellow for GFAP, cyan for IBA1).
4. **Quantification:** Segmented masks were analyzed in Fiji to calculate the % Area of GFAP+ and IBA1+ cells, for quantitative comparison of gliosis between sham- and MMT-treated animals.

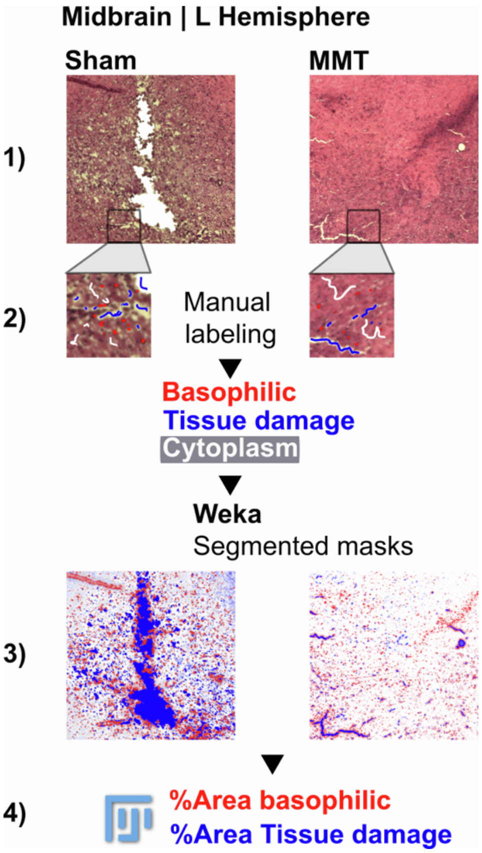

Supplemental Figure 3 (related to Figure 4). Weka-based segmentation workflow for quantifying tissue damage in H&E-stained brain sections.

Representative machine learning-assisted image analysis pipeline for quantifying histopathological changes in H&E-stained brain tissue following LPS injection and sham or MMT treatment.

1. **Raw Image**: Brightfield images of the H&E-stained left hemisphere midbrain from MMT (left) or sham (right) were acquired, showing cellular and structural features of brain tissue.
2. **Manual labeling & Training**: Selected ROIs were manually annotated to distinguish dark basophilic regions (indicating immune cell infiltration, labeled in red) from intact

pink cytoplasm (labeled in white) and tissue voids (indicating degeneration, labeled in blue). These annotations trained the Weka segmentation classifier.

3. **Weka Segmentation**: The trained model was applied to the entire image to automatically segment and color-coded tissue classes.
4. **Quantification**: Segmented masks were analyzed in Fiji to calculate the %Area basophilic and %Area tissue damage, for quantitative comparison of histopathological changes between sham- and MMT-treated animals.

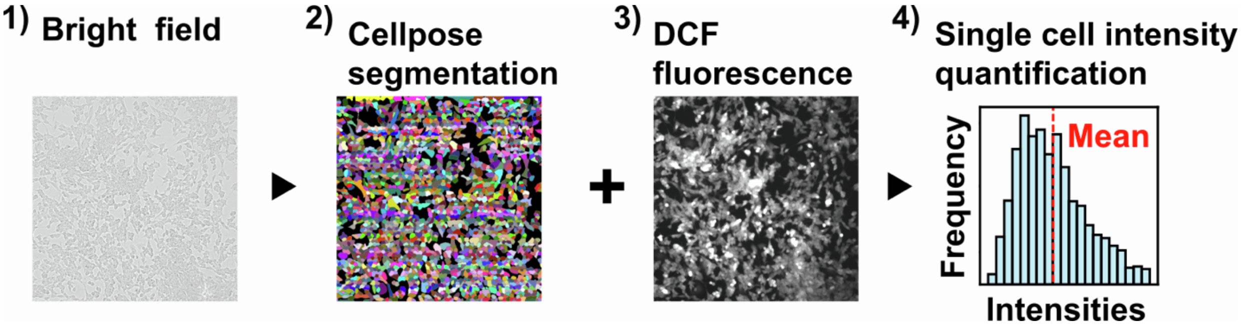

Supplemental Figure 4 (related to Figures 2E and 5). Cellpose-based analysis workflow for quantifying single-cell fluorescence intensity in cell populations.

Representative workflow for single-cell analysis of cellular fluorescence. Example shown for DCF fluorescence quantification in SH-SY5Y cells.

1. **Image Acquisition**: Brightfield and fluorescence images of cultured cells were acquired at 20x.
2. **Cell Segmentation**: The brightfield image was processed using the Cellpose deep learning Cyto3 model to automatically generate unique segmentation masks for each individual cell in the analyzed image.
3. **Single-cell intensity quantification**: The segmentation mask, containing a unique ID for each cell, was overlaid on the fluorescence image to calculate DCF fluorescence intensity for each cell.
4. **Population analysis**: The distribution of fluorescence intensities across the population is shown. Mean intensities from each independent experiment were calculated and reported as mean ± SD.

Microgliosis - IBA1

Brain Region	Hemisphere	Marker	Sham (%Area)	MMT (%Area)	Δ Change (%)	p-value	Significance
Cortex	L	IBA1	0.23 ± 0.55	0.11 ± 0.55	-51	0.55	ns
Cortex	R	IBA1	0.12 ± 0.33	0.16 ± 0.33	39	0.71	ns
Subcortex	L	IBA1	5.53 ± 0.93	2.88 ± 0.93	-48	0.33	ns
Subcortex	R	IBA1	2.61 ± 0.57	2.18 ± 0.57	-17	0.55	ns
Midbrain	L	IBA1	9.52 ± 3.51	2.02 ± 3.51	-79	0.02	*
Midbrain	R	IBA1	5.29 ± 4.90	1.01 ± 4.90	-81	0.01	*

Astrogliosis - GFAP

Brain Region	Hemisphere	Marker	Sham (%Area)	MMT (%Area)	Δ Change (%)	p-value	Significance
Cortex	L	GFAP	0.62 ± 0.12	0.71 ± 0.12	15	0.89	ns
Cortex	R	GFAP	0.48 ± 0.61	1.05 ± 0.61	1.2e2	0.51	ns
Subcortex	L	GFAP	1.99 ± 0.74	0.99 ± 0.74	-51	0.44	ns
Subcortex	R	GFAP	0.56 ± 0.70	1.05 ± 0.70	87	0.44	ns
Midbrain	L	GFAP	3.98 ± 2.05	0.40 ± 2.05	-90	0.12	ns
Midbrain	R	GFAP	2.43 ± 0.92	0.78 ± 0.92	-68	0.36	ns

Hemisphere Summary

Marker	Hemisphere	Sham (%Area)	MMT (%Area)	Δ Change (%)	p-value	Significance
IBA1	Left	5.10 ± 4.66	1.67 ± 1.42	-67.2	0.290	ns
IBA1	Right	2.67 ± 2.59	1.12 ± 1.01	-58.1	0.387	ns
GFAP	Left	2.20 ± 1.69	0.70 ± 0.29	-68.1	0.205	ns
GFAP	Right	1.16 ± 1.10	0.96 ± 0.16	-16.7	0.778	ns

Supplemental Table 1 (related to Figure 3). Quantitative immunohistochemistry of glial markers (GFAP and IBA1) across brain regions.

This table reports the mean percentage area (%Area) of GFAP (astrocytic) and IBA1 (microglial) immunoreactivity across cortical, subcortical, and midbrain regions in Sham and MMT groups (n = 3 rats per group). Data are presented as mean ± SD and percent change (Δ%) between matched regions of sham and MMT conditions.

Basophilic Enrichment

Brain Region	Hemisphere	Sham (%Area)	MMT (%Area)	Δ Change (%)	p-value	Significance
Cortex	Left	15.14 ± 6.65	2.53 ± 1.37	-83	0.013	*
Cortex	Right	9.13 ± 6.96	5.10 ± 0.89	-44	0.26	ns
Subcortex	Left	6.69 ± 2.39	2.16 ± 1.11	-68	0.029	*
Subcortex	Right	4.72 ± 1.07	1.99 ± 1.47	-58	0.038	*
Midbrain	Left	12.63 ± 0.79	0.62 ± 0.55	-95	0.002	**
Midbrain	Right	5.25 ± 2.18	1.78 ± 1.32	-66	0.041	*

Tissue Degeneration

Brain Region	Hemisphere	Sham (%Area)	MMT (%Area)	Δ Change (%)	p-value	Significance
Cortex	Left	12.02 ± 6.61	0.36 ± 0.26	-97	0.008	**
Cortex	Right	9.97 ± 9.02	1.74 ± 1.63	-83	0.036	*
Subcortex	Left	2.52 ± 2.93	0.41 ± 0.27	-84	0.041	*
Subcortex	Right	1.29 ± 1.68	0.45 ± 0.37	-65	0.071	ns
Midbrain	Left	6.08 ± 3.91	0.16 ± 0.12	-97	0.004	**
Midbrain	Right	1.57 ± 1.92	0.42 ± 0.35	-73	0.049	*

Combined Summary)

Marker	Hemisphere	Sham (%Area)	MMT (%Area)	Δ Change (%)	p-value	Significance
Basophilic Enrichment	Left	11.48 ± 5.29	1.88 ± 1.24	-83.6	0.000	**
	Right	6.47 ± 4.92	3.11 ± 2.31	-52.0	0.082	ns
Tissue Degeneration	Left	6.76 ± 5.96	0.41 ± 0.27	-94.0	0.006	**
	Right	5.70 ± 16.14	0.85 ± 1.16	-85.1	0.382	ns

Supplemental Table 2 (related to Figure 4). Quantitative histopathology of H&E-stained brain regions.

This table reports the mean percentage area (%Area) of dark nuclei (immune cell infiltration) and voids (tissue damage) measured from H&E-stained brain sections in Sham and MMT groups, in cortical, subcortical, and midbrain regions of sham and MMT groups. Data are presented as mean ± SD and percent change (Δ%) between matched regions of sham and MMT conditions.
